# Primary Epiploic Appendagitis: A Case Report

**DOI:** 10.7759/cureus.14060

**Published:** 2021-03-23

**Authors:** Alpaslan Mert, Emre Mırcık

**Affiliations:** 1 School of Health Sciences, Beykent University, Istanbul, TUR; 2 Health Management, Private Hurrem Sultan Hospital, Istanbul, TUR

**Keywords:** acute abdomen, computed tomography, exploratory laparotomy, primary epiploic appendagitis (pea)

## Abstract

Primary epiploic appendagitis (PEA) is a rather uncommon and self-limiting cause of acute abdomen managed conservatively. Overlapping clinical features with other common causes of acute abdomen usually requiring surgical intervention, and rare occurrences have led to misdiagnosis of the condition and unnecessary surgical intervention. However, with identification of definite characteristic features on imaging (computed tomography [CT] scan) has led to easier diagnosis and avoidance of exploratory laparotomy. Here we present a case of PEA in a 34-year-old otherwise healthy Caucasian male with a chief complaint of acute left-sided abdominal, flank and inguinal pain with diarrhea. Laboratory investigation reports were more or less within normal limits; CT scan confirmed the diagnosis of PEA. The patient was managed successfully with an oral antibiotic and a non-steroidal anti-inflammatory drug. CT scan should be done in cases of acute abdomen (if not absolutely contraindicated) for confirmation of diagnosis, as in our case CT scan helped in confirmation of diagnosis of PEA and thus avoided unnecessary surgical intervention. However, with the current advances in radiological tools, correct diagnosis of acute abdomen has become a lot easier, leading to timely surgical intervention and also at the same time avoidance of unnecessary exploratory laparotomy. Again, with documentation of specific characteristic radiological features of PEA, diagnosis of PEA has become much easier. After careful correlation among clinical, radiological, and laboratory findings, diagnosis of PEA was confirmed. The patient was managed conservatively at home with the advice of plenty of fluid intake and bed rest. Furthermore, he was prescribed an oral antibiotic (ciprofloxacin) and a non-steroidal anti-inflammatory drug (ibuprofen) empirically for seven days to prevent further complications like adhesions, bowel obstruction, intussusception, peritonitis, and local abscess formation. The patient recovered completely (the symptoms and signs resolved clinically) after one week. To conclude, it can be said, although rare in occurrence and lacking in specific presenting features, diagnosis of PEA has become easier with imaging techniques like CT scan and magnetic resonance imaging (MRI); thus, with prior awareness regarding this disease among physicians, unnecessary surgical interventions can be avoided.

## Introduction

Epiploic appendages are tiny, mobile fatty pouch-like structures (approximately 50-100 in number in adults) arising from the peritoneum and typically located on the outer part of the tenia coli (from sigmoid colon to cecum) [1]. With pedunculated nature, broad base, and being drained by single vein, these appendices are susceptible to torsion, infarction (both hemorrhagic and ischemic), thrombosis, and inflammation leading to primary epiploic appendagitis (PEA) [1-4]. Torsion and inflammation are the most common causes of PEA [2,5].

PEA is one of the rare causes of acute abdomen. The common sites of PEA are at the sigmoid colon and at the ileocecal regions with localized pain often leading to misdiagnosis of the case as diverticulitis and acute appendicitis, respectively [6].

Although it is a self-limiting condition where most of the patients recover with conservative management, because of its rare occurrence, and non-specific clinical features (overlapping other causes of acute abdomen like acute appendicitis, diverticulitis, cholecystitis, and obstructed hernia), there is a high possibility of misdiagnosing the condition and performing unnecessary laparotomy [1,5].

However, with the current advances in radiological tools, correct diagnosis of acute abdomen has become a lot easier, leading to timely surgical intervention and also at the same time avoidance of unnecessary exploratory laparotomy. Again, with documentation of specific characteristic radiological features of PEA, diagnosis of PEA has become much easier. Here, we present a case of PEA in an otherwise healthy male patient who was managed successfully with conservative approach.

## Case presentation

A 34-year-old Caucasian man, manual worker by profession (with body mass index of 26 kg/m^2^), presented to the general surgery department in our hospital with lower abdominal pain with bloating. His VAS (visual analog scale) pain score was 3 out of 10. Abdominal examinations were normal and per rectal clinical examination findings were hemorrhoid. The patient was diagnosed with hemorrhoids and gastritis and was prescribed proton pump inhibitor (pantoprazole) and otilonium bromide with simethicone for gastritis, and capillary stabilizing agent (essin) for hemorrhoids.

The patient attended the family medicine outpatient department of our hospital within four days of initial visit. This time the patient had pain in the left lower quadrant abdomen, left flank, left inguinal region along with bloating, diarrhea, passage of black stool, and itching and pain in the anal area. VAS pain score was 7 out of 10. Abdominal pain was not related with eating and defecation. He did not have nausea, vomiting, fever, chills, skin rash, joint pain, dysuria, hematuria, loss of weight, and trauma to the abdomen. There was no recent travel history or contact with sick person. Also, there was no history of illicit drug abuse. He denied presence of any significant stressors at home or at work.

On further enquiry, the patient reported intermittent abdominal pain and stomach bloating for the past seven to eight years. He was a smoker (smoked one and a half packs in a day). There was no personal or family history of irritable bowel syndrome or inflammatory bowel disease; however, his mother underwent surgery for colon cancer.

On physical examination, the patient had tenderness and pain in the left iliac fossa associated with abdominal guarding, suggestive for diverticulitis. There was no pulsatile or palpable mass. All of his vital signs were within normal limits. Physical examination was otherwise unremarkable.

Laboratory investigations revealed total leukocyte count (TLC) 6.4×10^3^/µL, hemoglobin: 15.1 g/dL, hematocrit: 43.6%, red blood cell level: 5.4×10^6^/mm^3^, and platelet: 2.33×10^5^/µL.

Urine analysis report was within normal limits.

Fecal occult blood was positive for transferrin and hemoglobin.

After consulting with radiologist, computed tomography (CT) scan of the abdomen and pelvis without contrasts was performed.

**Figure 1:**
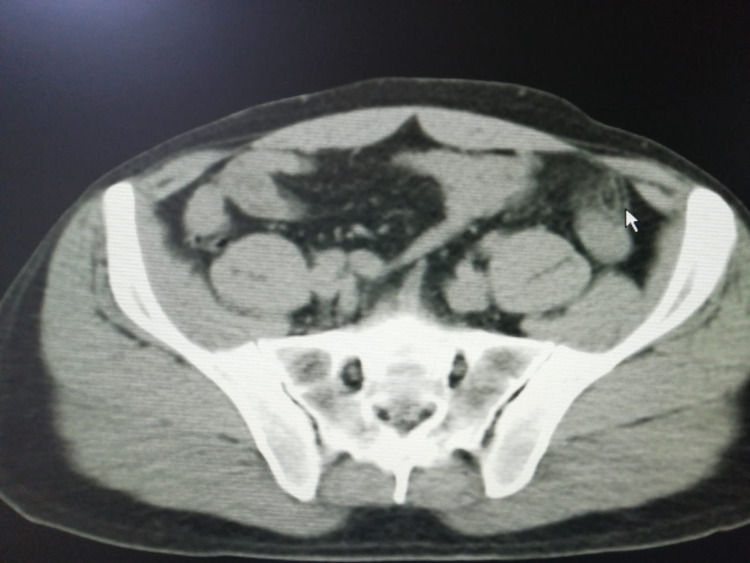
Ovoid lesion 14x7 mm in diameter, which reached out to anterior from the meso of colon, in lower tip of descending colon in the left lower quadrant, and inflammatory changes in the adjacent fat tissue.

The abdominal CT scan (without contrast) (with sequential sections of 5 mm) revealed presence of an ovoid lesion 14x7 mm in diameter, extending anteriorly from the mesocolon to the lower tip of the descending colon in the left lower quadrant; also, there were inflammatory changes in the adjacent fat tissue (suggestive of primary epiploic appendagitis) (Figure 1).

After careful correlation among clinical, radiological, and laboratory findings, diagnosis of PEA was confirmed.

The patient was managed conservatively at home with the advice of plenty of fluid intake and bed rest. Furthermore, he was prescribed oral antibiotic (ciprofloxacin) and non-steroidal anti-inflammatory drug (ibuprofen) empirically for seven days to prevent further complications like adhesions, bowel obstruction, intussusception, peritonitis, and local abscess formation.

The patient recovered completely (the symptoms and signs resolved clinically) after one week.

## Discussion

Epiploic appendagitis was first described in the year 1956 by Dockerty et al. [7]. It is of two types, primary (PEA) and secondary. PEA (very rare in occurrence) usually occurs due to inflammatory changes following torsion of the appendages or occlusion of the venous drainage, whereas secondary epiploic appendagitis occurs due to secondary inflammation in the adjacent structures like appendicitis, diverticulitis, pancreatitis, and postsurgical adhesions [8].

Although PEA can affect people of any age group, people in the fourth and fifth decades of life are the most commonly affected [3]. Males are more susceptible to it with obesity being one of the common risk factors of PEA, especially with people who have lost significant amount of weight within a short span of time; also, people who do strenuous exercise are more commonly affected [5,8]. In our case, the patient was overweight if not obese (body mass index 26 kg/m^2^). The diagnosis of PEA is not straightforward as it presents with nonspecific and overlapping clinical features like other common causes of acute abdomen (acute-onset sharp pain in the left or right quadrant of the lower abdomen, which might worsen with movement or cough) like acute appendicitis and diverticulitis. Also, because of its rare occurrence, it is highly possible that the surgeon might not consider it as a differential diagnosis.

Regarding presenting symptoms of PEA, Chen and his colleagues reviewed the presenting symptoms and CT scan findings of 21 patients of EA (5). Abdominal pain without any accompanying fever was the most common presenting feature in all those patients [5]. However, other than acute-onset abdominal pain, anorexia, nausea, vomiting, diarrhea, and even constipation might sometimes occur. Similar to the above findings, our patient also presented with acute-onset pain in the abdomen without any fever along with diarrhea with passage of black stool and bloating.

On clinical examination, abdominal tenderness and guarding are common findings; in our patient, we had similar findings [9]. However, other not so common signs like rebound tenderness (elicited only in 25% cases) and palpable inflammatory mass (palpable in 10-30% of the cases) were not seen in our patient [9].

There are no specific laboratory findings for PEA other than nonspecific rise in inflammatory markers like C reactive proteins and occasional leucocytosis; usually other blood parameters remain within normal limits [4,5]. In our patient, even the leucocyte count was within normal range (TLC, 6.400/mm^3^)

Thus, the lack of specific clinical signs and symptoms, and laboratory findings and rarity of the disease makes the diagnosis of PEA very much challenging. Often patients of PEA are misdiagnosed as acute appendicitis or diverticulitis depending upon the site of pain in the abdomen. In our case because of left-sided pain in the abdomen, initial diagnosis after clinical examination was that of diverticulitis.

In earlier days without radiological support (CT scan or color Doppler ultrasound scan), PEA was most commonly diagnosed during exploratory laparotomy. However, fortunately, nowadays with the help of CT scan and color Doppler ultrasound, diagnosis of PEA has become easier.

PEA has typical characteristic features on CT scan with regard to the location, size, and density of the lesion. Singh and his colleagues described the spectrum of contrast-enhanced CT scan findings in 50 patients diagnosed with acute epiploic appendagitis (10). As per their study the most commonly affected site is sigmoid colon (62%) followed by descending colon (18%), and ascending colon being the least commonly affected site (8%) [10]. In our patient, CT scan revealed that the lesion was located at the lower part of descending colon.

Again, Singh and his colleagues also described in their study that usually (in 86% of the cases) the fatty portion of the lesion measures between 1.5 and 3.5 cm with inflammation in the surrounding region [10]. In our case the lesion had an overall size of 14×7 mm with inflammatory changes in the adjacent tissue.

Magnetic resonance imaging (MRI) is not routinely done in PEA; however, because of the lack of risk of radiation exposure it is preferred in children and pregnant women [11].

Despite the specific radiological features of PEA on CT scan, radiologists might miss the diagnosis because of lack of prior knowledge and exposure to these characteristic findings.

PEA being a self-limiting disease is managed conservatively with a better understanding of the disease, and conservative management is established as the treatment of choice [4,5,12].

Also, unlike earlier days, because of better imaging techniques (CT scan and MRI), accurate diagnosis has led to avoidance of unnecessary surgery in PEA patients.

Usually the symptoms of PEA relieve within one to four weeks with conservative management (9). Ozdemir and colleagues published a case series on PEA; they reported that symptoms of PEA resolved within three weeks with conservative treatment and they remained symptom free during the seven-week follow-up period [13].

Our patient was managed conservatively with antibiotic (to avoid complications) and oral non-steroidal anti-inflammatory drugs. Although oral anti-inflammatory drugs are recommended for four to seven days for pain, antibiotic is usually not recommended unless there is fear of complications.

Surgical intervention is rarely required in patients who do not respond to conservative management or develop complications like intussusception, abscesses, and intestinal obstruction.

## Conclusions

To conclude, it can be said, although rare in occurrence and lacking in specific presenting features, diagnosis of PEA has become easier with imaging techniques like CT scan and MRI; thus, with prior awareness regarding this disease among physicians, the unnecessary surgical interventions can be avoided.
